# Team-Based Simulation for Medical Student Handoff Education

**DOI:** 10.15766/mep_2374-8265.10486

**Published:** 2016-10-21

**Authors:** Alanna Higgins Joyce

**Affiliations:** 1 Assistant Professor, Department of Pediatrics, Northwestern University The Feinberg School of Medicine

**Keywords:** Feedback, Simulation, Handoffs, Debriefing, Patient Handoff, Small-Group Learning

## Abstract

**Introduction:**

Good handoffs require teamwork, clear communication, a cognitively safe environment, and a good understanding of the patient's medical needs. Complex tertiary care training institutions require multiple handoffs over a patient's time in the hospital. Medical students need better handoff education. We designed a handoff training exercise using a simulated patient care environment for students to practice and observe multiple handoffs over time.

**Methods:**

An initial large-group didactic session provides direct instruction on handoffs. In small groups, students are subsequently assigned roles as individual physicians in a chain of providers during a simulated patient hospitalization over several days, with an additional student as an observer. Blinded to any prior discussion, student physicians sequentially give and receive handoffs about the patient from a previous physician as their simulated hospital course evolves. The observer shares his or her insights, and a large-group structured debriefing exercise follows.

**Results:**

In both 2015 and 2016, we implemented this session with a cohort of 30 fourth-year medical students. Most recently we implemented this with chief residents, and a group of 20 third-year pediatric clerkship students. We reviewed a selection of the discussion guides and found reporters/ observers noted that participants stated the primary problem 95% of the time (19 of 20 handoffs), the patient acuity 90% of the time (18 of 20 handoffs), and a clear contingency plan 85% of the time (17 of 20 handoffs).

**Discussion:**

We found students initially to be preoccupied with making correct clinical decisions instead of giving effective handoffs, and consequently we clarified details and adjusted clinical information to be more transparent. Results suggest the session achieves our goals of using a structured handoff method to communicate higher-level information and debrief effectively with peers. We envision this activity to be applicable to teaching handoffs to residents, nurses, and other health care professionals, as well as possibly in diverse clinical environments or in collaboration with outpatient providers.

## Educational Objectives

By the end of the session, learners will be able to:

1. Use a structured handoff method to give a simulated handoff in a longitudinal patient care environment.
2. Apply higher-level communication skills related to patient handoffs to transfer a simulated patient's primary problem, acuity, and contingency plan to a receiving provider.
3. Give peer-to-peer feedback during a structured debriefing exercise to encourage self-reflection in a cognitively safe environment.

## Introduction

One of the great challenges of medicine is that “at some point in the day or the week or the call cycle … physicians … must go home, and care must be handed over to someone else. This is a biologic and logistic imperative.”^1^ As a result, modern patient care, particularly in academic institutions, is delivered by multiple providers with varying degrees of knowledge of their patients.^2^ Handoffs between providers, in which control of and responsibility for a patient pass from one health professional to another, are increasing.^3^ There are no easy solutions: Lengthy shifts have been associated with an increase in serious medical errors,^4^ while flawed handoffs create the potential for medical errors and serious health consequences for patients.

Many metrics have been developed to examine and improve individual handoffs. Simulated handoff experiences, checklists, and mnemonics all have been shown to reduce mistakes.^5,6^ However, although a singular handoff may be excellent, the successful secondary transmission of patient information is crucial for continued high-quality health care delivery. We believe studying more than one handoff in sequence is important; like a game of telephone, nuance or substance may be lost the further one is from the initial successful handoff. There are not currently many educational exercises that allow several sequential handoffs of one patient to be practiced formally in a compressed time period among peers.

Implementing structured handoff environments and focused handoff education for medical residents has been associated with a significant reduction in medical errors and preventable adverse events among hospitalized children.^7^ While handoffs among medical residents have been scrutinized in light of their lengthy work hours, we know little about how medical students learn and execute handoffs. A 2014 study^8^ on handoff practices in undergraduate medical education reported that few medical students are taught handoff competencies in medical school. Specifically, in a national survey, only 15% of clerkships had structured handoff training; however, 93% reported that subinterns (students in their fourth year) performed handoff activities.^8^

Simulation has been shown to enhance medical education and teach complex skills. Students who participated in a simulation exercise performed better on a knowledge-based test and reported increased comfort and perceived competence in their clinical approach.^9^ Team-based training has also been proven effective in health care; the Teamwork Mini-Clinical Evaluation Exercise focuses on six collaborative competencies in health care^10^ through direct observation and discussion exercises.

MedEdPORTAL contains activities related and adjunctive to our session. The most-established similar tool is the Medical Student Workshop component of the I-PASS Handoff Curriculum.^11^ This includes a didactic session devoted to a standardized approach to the handoff process and team training in structured communication techniques, followed by two handoff simulation exercises featuring small-group role-playing activities under faculty or resident supervision. Completing both the didactics and simulation requires 90 minutes, in contrast to our session, which can be completed in 60 minutes. As well, while our material may be used unaccompanied, it is recommended that the I-PASS Medical Student Workshop be used within the larger suite of materials applicable to residents, faculty, and staff in a hospital-wide adoption of the I-PASS campaign.

Several additional publications relate to our project. One role-play activity specifically for medical students involves viewing an instructional video and then practicing postdischarge communication with peers.^12^ Another handoff simulation for residents involves a case on pulmonary embolus followed by a structured debriefing; residents are expected to manage the care of multiple patients after receiving a sign-out from the instructor.^13^ A daylong curriculum for senior medical students approaching graduation uses small-group discussion, didactic presentations, role-playing, and facilitated debriefing to teach multiple skills geared toward successful transition to internship.^14^ It includes handoff education but also features formulating assessments and plans, identifying tasks, and answering pages. Whereas all these tools involve simulated handoffs, none focus specifically on handoff education solely for medical students with a simulation involving many sequential cases over time.

Both students and teachers know how to operate within a top-down, content-driven approach that rewards strategic learning. In contrast, our session allows students to take ownership of their learning and reflect on their challenges among peers. Learning is better when students are given ownership over a decision, have creativity, and are learning novel, surprising, unusual content that is relevant to their daily life. Students must also be in an “emotionally literate environment” where they feel comfortable in themselves and with others.^15^ We believe our novel learning environment of simulation coupled with experiencing autonomy and working with peers facilitates student engagement, comfort, and skill building.

## Methods

We designed a handoff training exercise using a simulated patient care environment for students to practice and observe multiple handoffs over time. A large-group didactic session first provides direct instruction on handoffs. Students subsequently divide into smaller groups, with one student acting as observer and the remaining students assigned roles as physicians in a chain of caretakers during a simulated patient hospitalization over several days. Blinded to any prior discussion, students sequentially receive a handoff about the patient from the previous physician while being observed by a peer. The observer shares his or her insights, and a large-group structured debriefing exercise follows.

Ideal learners for the 60-minute session are medical students who have completed all junior clerkships and are preparing for subinternships or imminent graduation and residency training. The ideal group size is one leader and five to 20 students. The exercise may be done with as few as five students but typically is done with a group of 20–25, with multiple small groups divided into five students each.

The session should be held in a classroom for up to 20 students with slide presentation projection capability and modular furniture or multiple nearby spaces to facilitate small-group work. Facilitators should ensure that the audiovisual equipment in the room includes sound and projection capabilities to allow showing of the teaching video embedded in the presentation. Prior to the session, facilitators should prepare copies of the cases for distribution to each participant who is role-playing and copies of the discussion guide for all reporters/observers.

In Part 1 (15–20 minutes), the leader presents a short didactic session in which the exercise is described as confidential and ungraded. Direct instruction via the PowerPoint presentation (Appendix A) reviews skills related to high-quality handoffs and various techniques for handoffs. At the end of the presentation, the group views a short video^16^ designed to outline the many sources that contribute to communication failure. The video is followed with a think-pair-share^17^ activity where pairs of students discuss the answer to the question “What makes a good handoff?” Students are given 1 minute to think of and jot down a few qualities of good handoffs based on their viewing of the video. They then pair with one neighboring student (two are acceptable) and discuss their thoughts. Afterwards, one student from each pair then shares the pair's findings with the large group. The leader ends the didactic session with a review of the components of the I-PASS handoff technique^18^ using the provided handout (Appendix C).

In Part 2 (5–10 minutes), the students divide into small groups of exactly five students each, with the total number of groups dependent on the total number of students present for the session. Four of the students are assigned numbers 1 to 4, which correspond to various physician roles. The final student (number 5) is to act as observer/reporter. Students 1–4 are each simultaneously handed slips of paper containing different segments of information about the patient case (Appendix B) and are given a few minutes to review their own case segment. While Students 1–4 review the cases, the leader gathers all the observers/reporters and reviews expectations for recording during handoffs. The observer/reporter is to record the strengths and weaknesses and note whether the handoff giver states the patient's primary problem, acuity, and contingency plans.

In Part 3 (25–35 minutes), Students 3 and 4 are initially asked to leave the room. The observer/reporter (Student 5) remains throughout the exercise, using the included discussion guide (Appendix D) for recording during the activity. Student groups each perform four consecutive handoffs as follows:

• Student 1 (pediatrician) hands off to Student 2 (emergency department physician).
• Student 2 (emergency department physician) hands off to Student 3 (inpatient weekend provider).
• Student 3 (inpatient weekend provider) hands off to Student 4 (inpatient weekday provider).
• Student 4 (inpatient weekday provider) hands off to Student 1 (pediatrician).

Each handoff is timed by the leader to last no more than 6–8 minutes. Students are encouraged to base their handoff on techniques and principles reviewed previously. Sequential students (Students 3 and 4) enter when called to participate, blinded from previous handoffs by virtue of not being initially present in the room. They observe all subsequent handoffs after their own in order to best participate in the structured debriefing at the close of the activity. The leader circulates among the groups during the handoffs and observes so that he or she can later share overall feedback in the large-group debriefing.

In Part 4 (5–10 minutes), Student 5, the observer/reporter, shares his or her findings with the small group. Specifically, he or she is asked to comment on communicating the patient's primary problem, acuity, and contingency plans for concerning situations. He or she may also discuss things done well and things missed, allowing the other students to reflect on their performance.

In Part 5 (10–20 minutes), each reporter/observer is asked to share findings from the small-group discussion with the larger group. This transitions to a large-group reflection in which all participants are asked to share their thoughts on the following questions:

• What were some things done well today?
• What were some things that could have been better?
• Was anything confusing or surprising to you?
• What is one big question or big idea you will take away from today?

## Results

We implemented our session in both 2015 and 2016, near graduation, with a cohort of 30 fourth-year medical students interested in pediatrics during a multidisciplinary week with several sessions dedicated to residency preparation. Most recently, our resource has been used at our institution by our chief residents, during a fellowship orientation, and with 20 third-year pediatric clerkship students while on the inpatient pediatrics service.

To evaluate our activity, we reviewed a selection of the completed discussion guides (Appendix D) written by the reporters/observers watching their peers give four sequential handoffs. The discussion guide contains discrete observations on each participant's handoff. A formative assessment to assist the leader in focusing instruction on the unique needs of each group of learners, the discussion guide is also meant to facilitate the structured debriefing exercise at the conclusion of each session. Student responses on 20 individual handoffs were reviewed. Reporters/observers noted that participants stated the primary problem 95% of the time (19 of 20 handoffs), the patient acuity 90% of the time (18 of 20 handoffs), and a clear contingency plan 85% of the time (17 of 20 handoffs).

The discussion guide also features a free-response area for each observer/reporter, which includes things done well and areas to improve. Selected comments in these areas included the following:

### Done Well

• “Nice to know she keeps her leg flexed and internally rotated.”
• “This is someone you need to watch for changes.”
• “Concise summary and good asking [receiver] for questions.”
• “Good situational awareness.”
• “Summarized important labs/images.”
• “Good chronology and timeline of important events.”
• “Mentioned needing follow-up.”
• “Very clear and descriptive.”
• “General appearance of patient was helpful.”
• “Follow-up plan for post-operative bleeding and follow-up blood culture.”

### Areas for Improvement

• “Slow down when reporting labs.”
• “[Didn't] give red flags.”
• “Told plan moving forward but didn't really talk about what to do for worsening [clinical status].”
• “[Needs] a clearer plan.”
• “Need more vitals/objective data.”
• “State acuity/status up front rather than at end.”
• “Could have shared more relevant history.”
• “Felt disjointed at times.”
• “Outline pediatrician's role in future management [on discharge].”
• “Incomplete story.”

## Discussion

Before active implementation, we piloted our session with a small group of 11 medical students early in their fourth year. The initial pilot session focused much more on medical decision-making and clinical decisions than the handoff. Medical students were preoccupied with clinical decisions (having the correct differential diagnosis) and management alternatives (choosing consultants, obtaining imaging) instead of giving effective handoffs. They reported confusion on details such as exact time line of events, choice of antibiotics, and logistics of hospital discharge.

Based on our outcomes, we clarified facts and adjusted clinical information to be more transparent. We eliminated any clinical decision-making or distracting details and expressly stated the diagnosis and management plan for the patient at each sequential handoff. The new case format, focusing less on clinical reasoning, better achieves our goal of active practice over multiple longitudinal patient handoffs in a supervised setting among peers. Subsequent implementation with additional groups has yielded similar feedback and more minor changes such as clarifying antibiotic use, updating the care time line in the hospital, and adding realistic details to the cases to enhance role-playing by participants.

Learners responded positively to our changes, and our results indicate our sessions to be effective in teaching their stated objectives. Students praised our commitment to directly teaching handoff skills and using a simulated patient case with multiple parts. They expressed relief at being given the answers to the case. They stated they could now focus solely on high-quality information transfer. They expressed gratitude at the opportunity to practice handoffs with their peers, particularly nearing medical school graduation and looking towards internship.

Results suggest that the session achieves our goals of using a structured handoff method to communicate higher-level information and debrief effectively with peers. The high frequency of handoffs that were said to contain a patient's primary problem, acuity, and contingency plan is encouraging. The candid free-response comments, both for things done well and for areas of improvement, indicate participants felt free to give their peers targeted feedback, both positive and negative, during the structured debriefing exercise. It is important to note that our data contain the individual views of the student observers/reporters. They are not ratings using a specific rubric or consultation with an expert. Providing the observers/reporters with a more precise measurement tool may be useful in the future.

There are several additional areas for improvement. Though particularly appealing to senior medical students, we envision our activity to be applicable to teaching handoffs to residents, fellows, registered nurses, and other health care professionals, as well as possibly in diverse clinical environments or in collaboration with outpatient providers. Additional case scenarios or training a standardized patient to role-play the initial patient presentation and ongoing hospital course would provide a more realistic experience for learners. Our next steps will be to create a tool to evaluate students in real handoff scenarios on the wards and obtain direct feedback from their resident teachers or preceptors.

## Appendices

A. Team-Based Simulation for Medical Student Handoff Education.pptx
B. Cases.docx
C. I-PASS.docx
D. Discussion Guide.docx All appendices are peer reviewed as integral parts of the Original Publication.
