# Supplementary material for: Team-Based Simulation for Medical Student Handoff Education
Source: MedEdPORTAL. 2016 Oct 21;12:10486. doi: 10.15766/mep_2374-8265.10486 (PMC6440419; doi:10.15766/mep_2374-8265.10486)
Supplement: Supplementary file 1 — A. Team-Based Simulation for Medical Student Handoff Education.pptx B. Cases.docx C. I-PASS.docx D. Discussion Guide.docx [file mep-12-10486-s001.zip › D. Discussion Guide.docx]

| **Handoff** | **Primary Problem stated?** | **Patient acuity stated?** | **Contingency plan clear?** | **Done Well** | **Areas to improve** |
| --- | --- | --- | --- | --- | --- |
| **Student #1** | □ Yes  □ No | □ Yes  □ No | □ Yes  □ No |  |  |
| **Student #2** | □ Yes  □ No | □ Yes  □ No | □ Yes  □ No |  |  |
| **Student #3** | □ Yes  □ No | □ Yes  □ No | □ Yes  □ No |  |  |
| **Student #4** | □ Yes  □ No | □ Yes  □ No | □ Yes  □ No |  |  |

Discussion Guide: Student #5, watch each handoff. Using the following table, record your comments as the activity progresses:
