# Supplementary material for: Team-Based Simulation for Medical Student Handoff Education
Source: MedEdPORTAL. 2016 Oct 21;12:10486. doi: 10.15766/mep_2374-8265.10486 (PMC6440419; doi:10.15766/mep_2374-8265.10486)
Supplement: Supplementary file 1 — A. Team-Based Simulation for Medical Student Handoff Education.pptx B. Cases.docx C. I-PASS.docx D. Discussion Guide.docx [file mep-12-10486-s001.zip › A. Team-Based Simulation for Medical Student Handoff Education.pptx]

## Slide 1
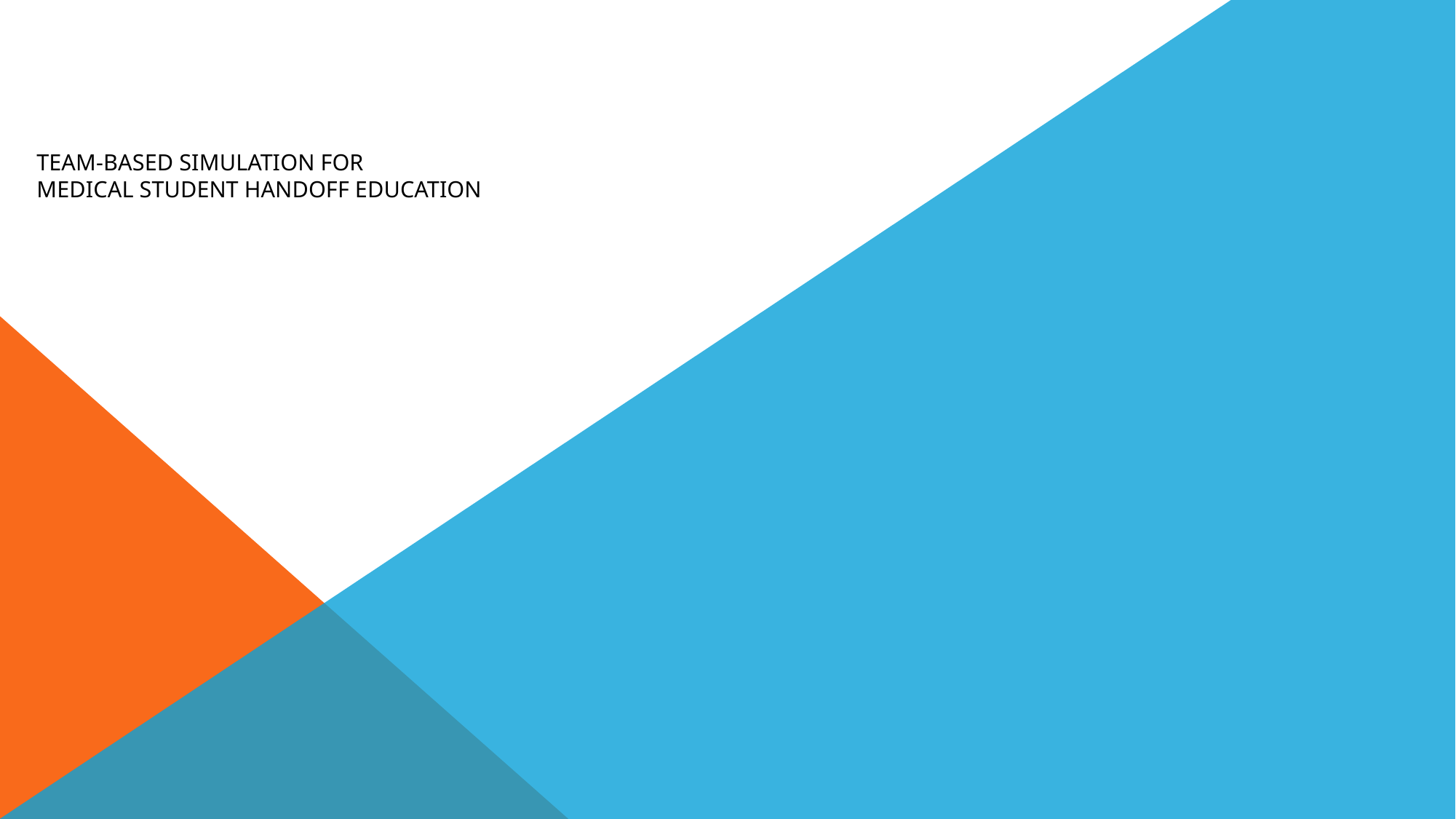

# Team-Based Simulation for Medical Student Handoff Education

## Slide 2
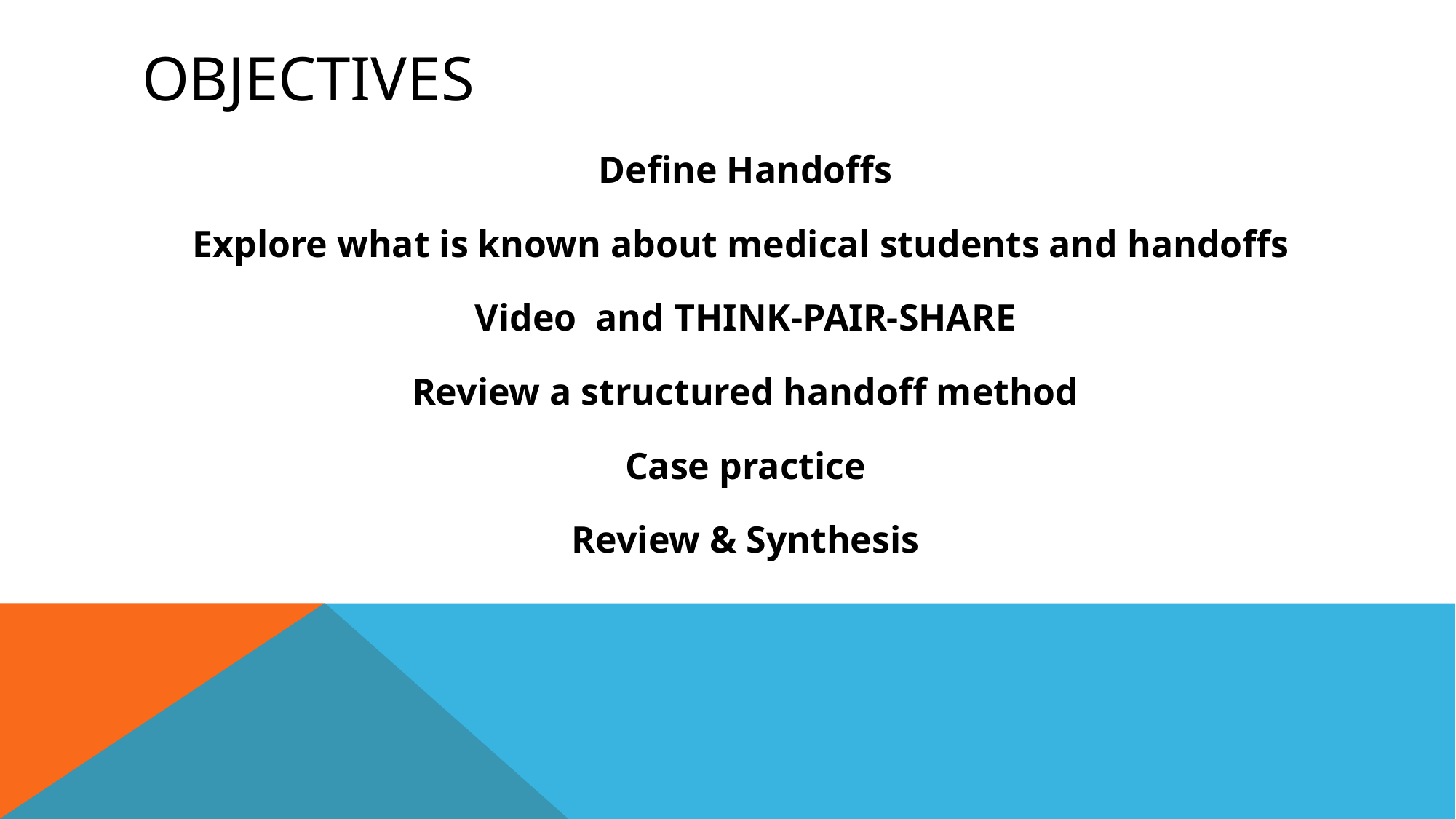

# Objectives
Define Handoffs
Explore what is known about medical students and handoffs
Video and THINK-PAIR-SHARE
Review a structured handoff method
Case practice
Review & Synthesis

## Slide 3
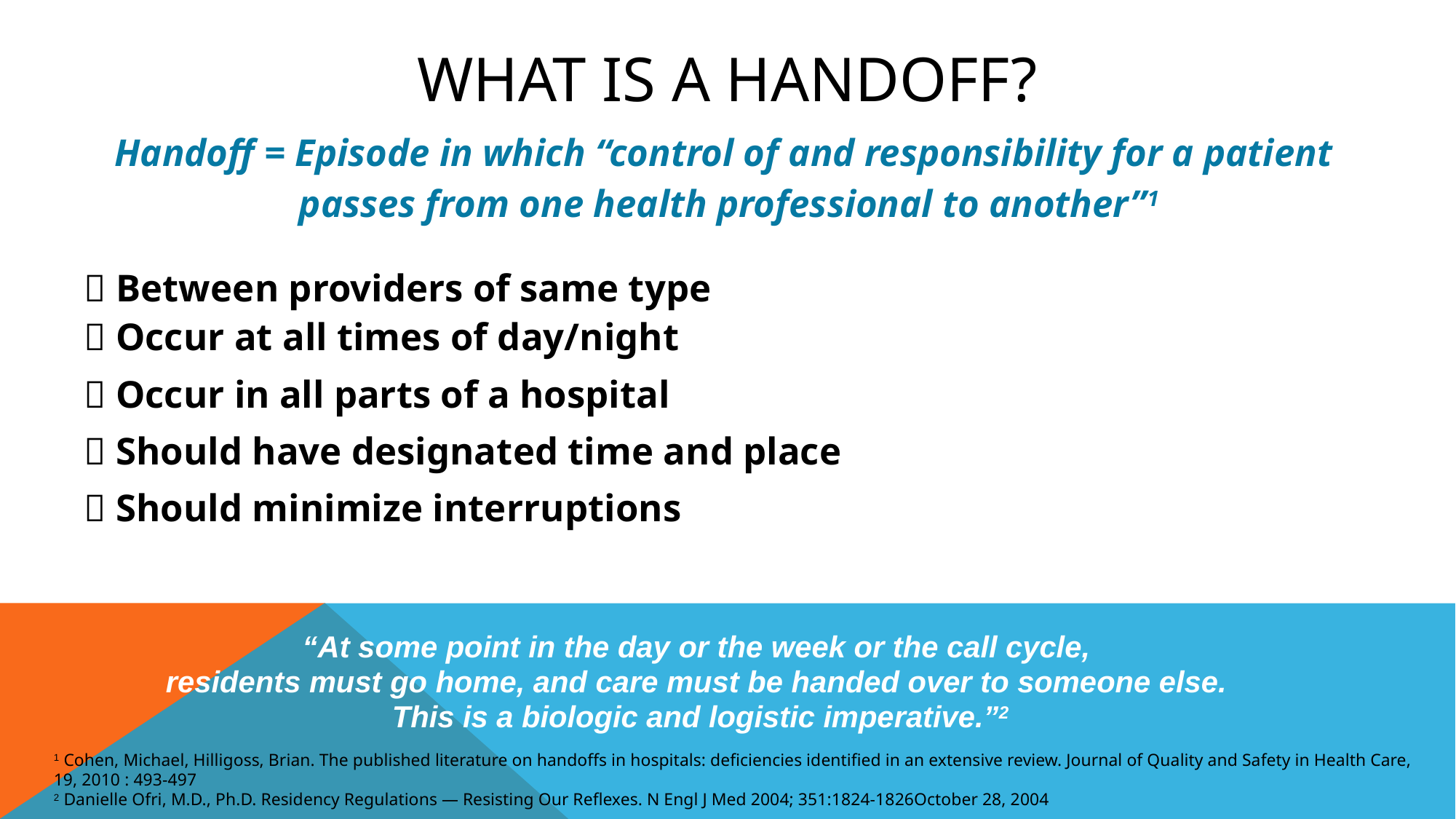

# What is a Handoff?
Handoff = Episode in which “control of and responsibility for a patient
passes from one health professional to another”1
 Between providers of same type
 Occur at all times of day/night
 Occur in all parts of a hospital
 Should have designated time and place
 Should minimize interruptions
“At some point in the day or the week or the call cycle,
residents must go home, and care must be handed over to someone else.
This is a biologic and logistic imperative.”2
1 Cohen, Michael, Hilligoss, Brian. The published literature on handoffs in hospitals: deficiencies identified in an extensive review. Journal of Quality and Safety in Health Care, 19, 2010 : 493-497
2 Danielle Ofri, M.D., Ph.D. Residency Regulations — Resisting Our Reflexes. N Engl J Med 2004; 351:1824-1826October 28, 2004

## Slide 4
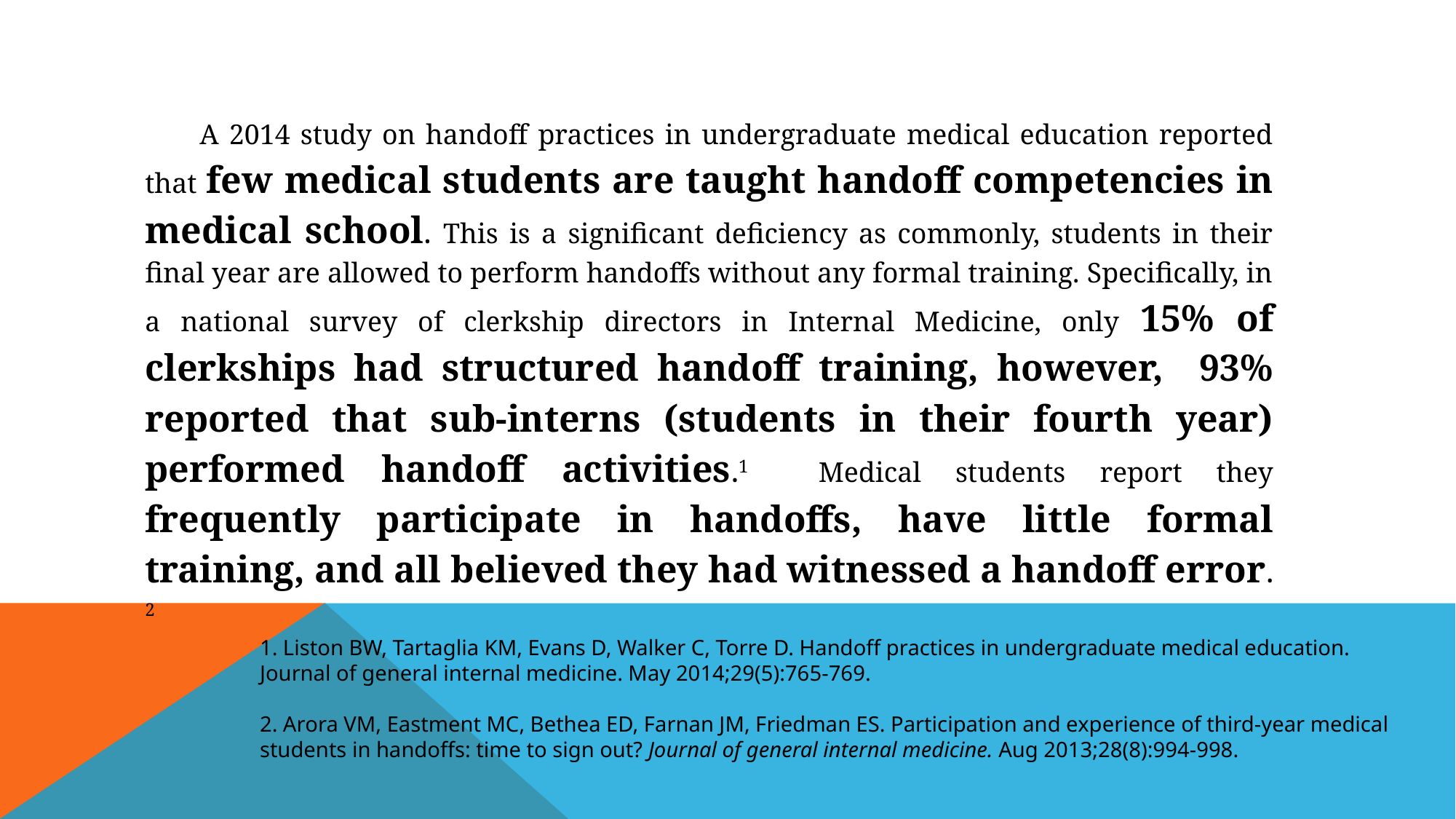

A 2014 study on handoff practices in undergraduate medical education reported that few medical students are taught handoff competencies in medical school. This is a significant deficiency as commonly, students in their final year are allowed to perform handoffs without any formal training. Specifically, in a national survey of clerkship directors in Internal Medicine, only 15% of clerkships had structured handoff training, however, 93% reported that sub-interns (students in their fourth year) performed handoff activities.1 Medical students report they frequently participate in handoffs, have little formal training, and all believed they had witnessed a handoff error. 2
1. Liston BW, Tartaglia KM, Evans D, Walker C, Torre D. Handoff practices in undergraduate medical education. Journal of general internal medicine. May 2014;29(5):765-769.
2. Arora VM, Eastment MC, Bethea ED, Farnan JM, Friedman ES. Participation and experience of third-year medical students in handoffs: time to sign out? Journal of general internal medicine. Aug 2013;28(8):994-998.

## Slide 5
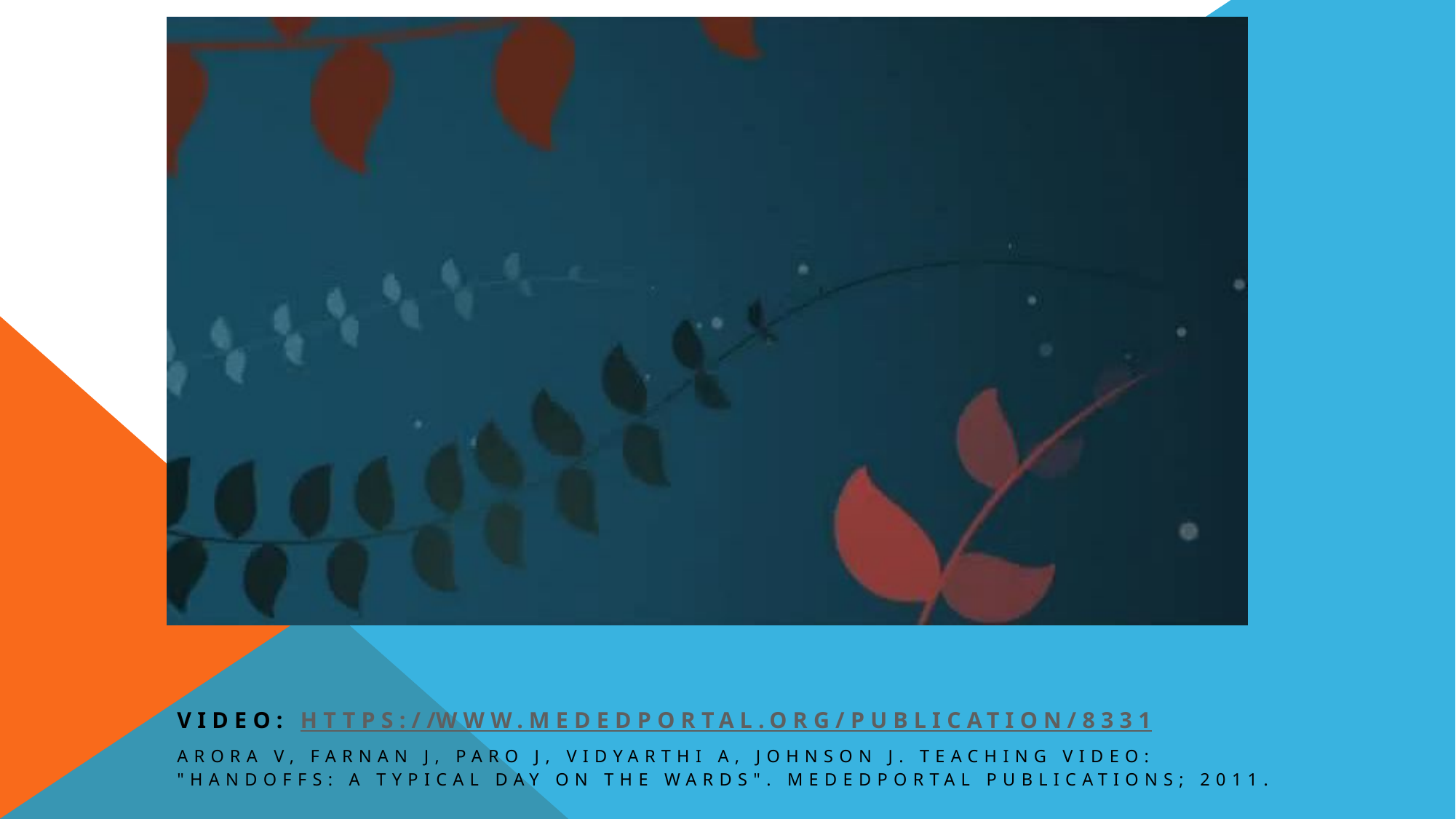

Video: https://www.mededportal.org/publication/8331
Arora V, Farnan J, Paro J, Vidyarthi A, Johnson J. Teaching Video: "Handoffs: A Typical Day on the Wards". MedEdPORTAL Publications; 2011.

## Slide 6
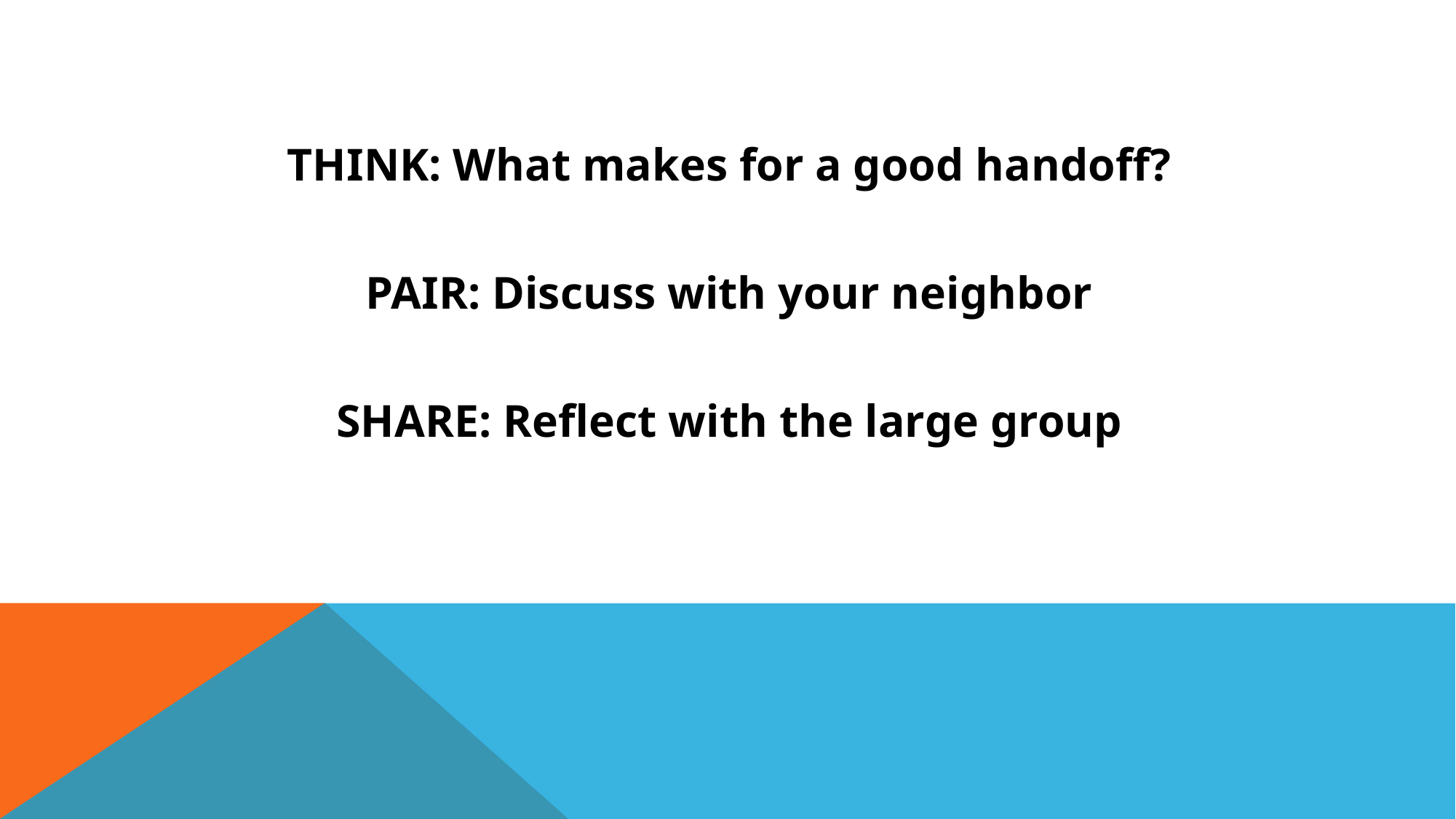

THINK: What makes for a good handoff?
PAIR: Discuss with your neighbor
SHARE: Reflect with the large group

## Slide 7
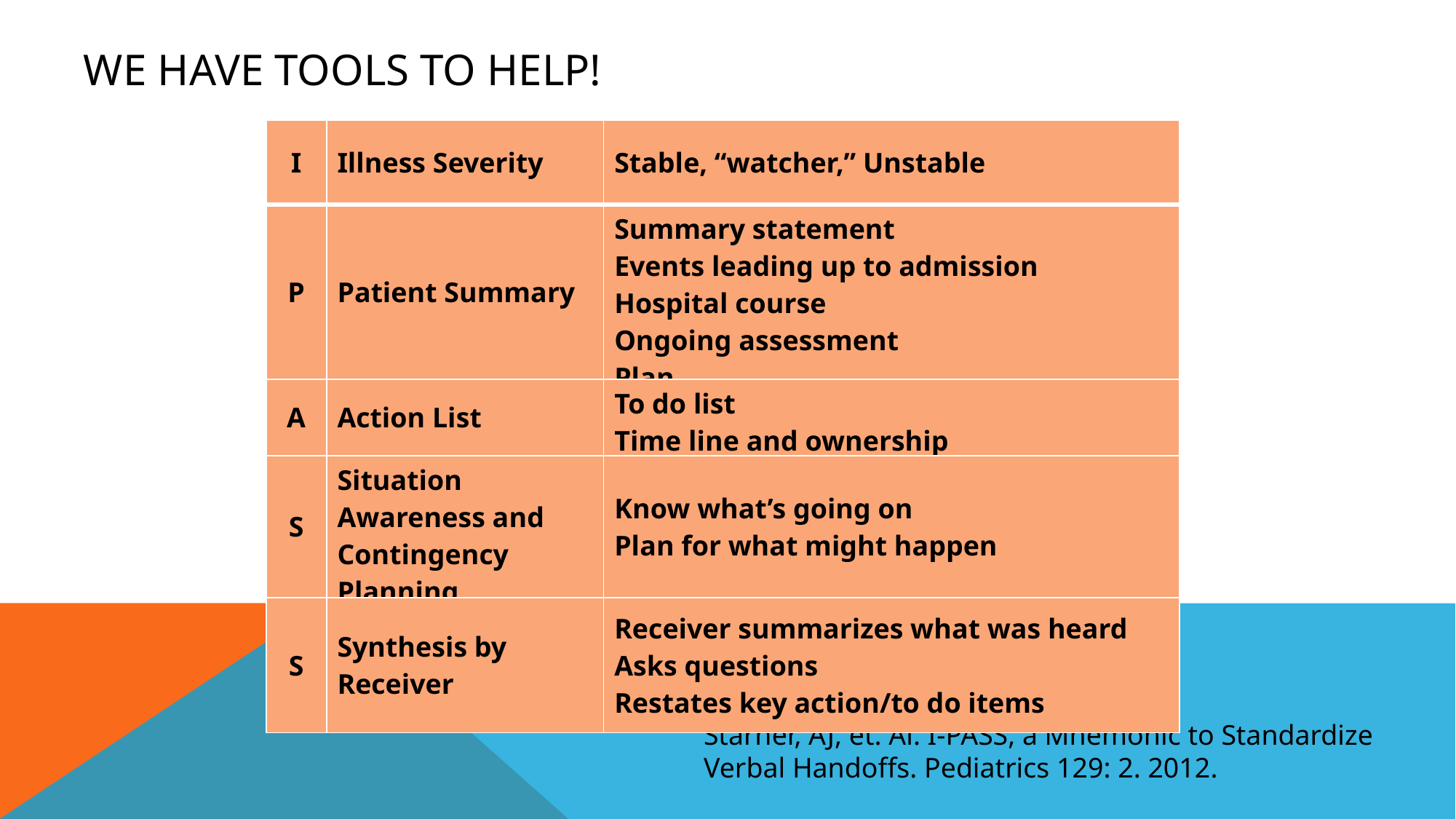

# We have tools to help!
| I | Illness Severity | Stable, “watcher,” Unstable |
| --- | --- | --- |
| P | Patient Summary | Summary statement Events leading up to admission Hospital course Ongoing assessment Plan |
| A | Action List | To do list Time line and ownership |
| S | Situation Awareness and Contingency Planning | Know what’s going on Plan for what might happen |
| S | Synthesis by Receiver | Receiver summarizes what was heard Asks questions Restates key action/to do items |
Starner, AJ, et. Al. I-PASS, a Mnemonic to Standardize Verbal Handoffs. Pediatrics 129: 2. 2012.

## Slide 8
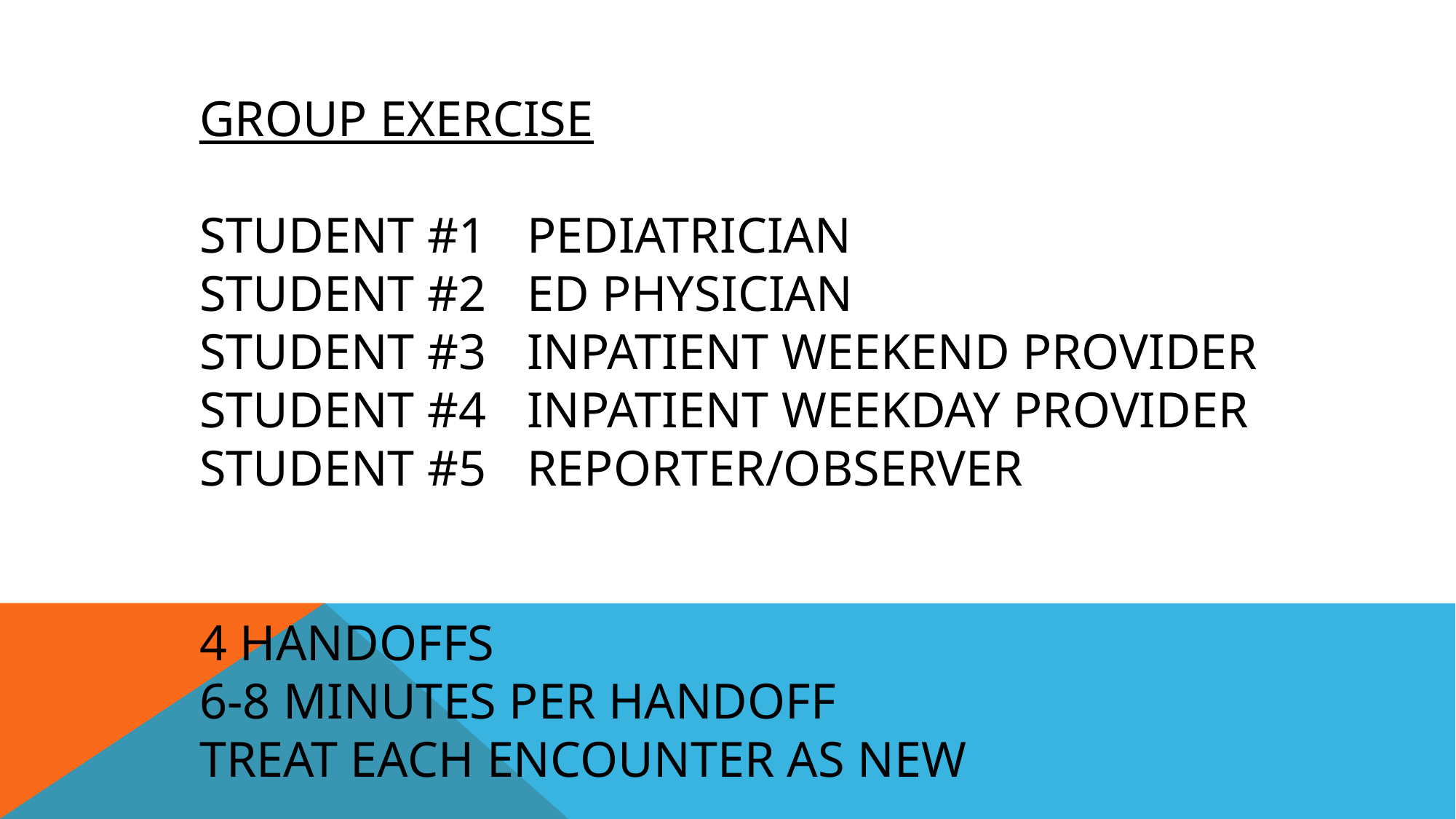

# Group Exercise Student #1	Pediatrician Student #2	ED Physician Student #3	Inpatient WEEKEND Provider Student #4	Inpatient WEEKDAY Provider Student #5	Reporter/Observer 4 Handoffs 6-8 minutes per handoff Treat each encounter as new

## Slide 9
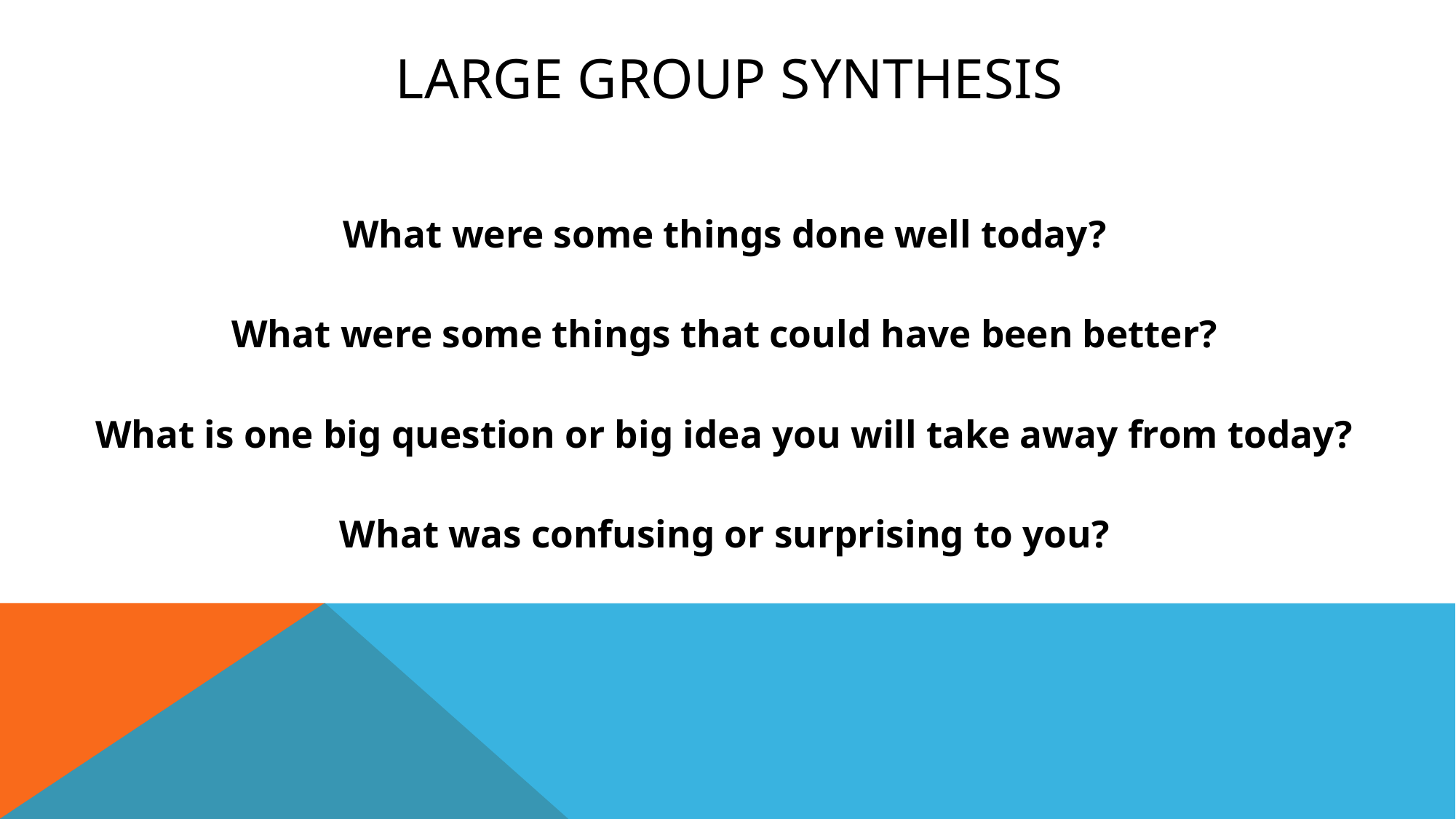

# Large Group Synthesis
What were some things done well today?
What were some things that could have been better?
What is one big question or big idea you will take away from today?
What was confusing or surprising to you?
