# Supplementary material for: Team-Based Simulation for Medical Student Handoff Education
Source: MedEdPORTAL. 2016 Oct 21;12:10486. doi: 10.15766/mep_2374-8265.10486 (PMC6440419; doi:10.15766/mep_2374-8265.10486)
Supplement: Supplementary file 1 — A. Team-Based Simulation for Medical Student Handoff Education.pptx B. Cases.docx C. I-PASS.docx D. Discussion Guide.docx [file mep-12-10486-s001.zip › B. Cases.docx]

**STUDENT #1 -> #2**

Thursday @ 10am. You are the Pediatrician seeing a sick office visit.

Chief Complaint: Limp

History of Present Illness: Olivia is a 2 year old with limp for two days and refusal to walk since this morning. Her parents think that her left leg that is bothering her. Yesterday she would walk slowly with a limp to get around, but today, she has refused to walk since this morning. She has been eating well. She hasn’t had fever. She and her 6 year old brother both had an upper respiratory infection about 2 weeks ago. There is no history of rash, vomiting, or diarrhea. Parents report no recent trauma. Olivia has not traveled anywhere recently or had any ill exposures.

Review of Systems: All systems reviewed and negative except as noted in the History of Present Illness.
Past Medical/Surgical History: No medical problems. No surgeries
Medications: None
Allergies: None
Social History: Lives at home with mom, dad, brother, and 2 cats.
Family History: Healthy parents.
Immunizations: Up to date.
Development: No concerns.

Physical Exam:
Vital Signs: temperature 37.9 (rectal), pulse 124, respirations 24, blood pressure 92/54, pulse oximetry 100%

Gen: Happy and playful while sitting on her parent’s lap, non-toxic
HEENT: normal
Neck: supple, no lymphadenopathy
CV: regular, normal S1/S2, no murmurs
Resp: clear bilaterally, good expansion and aeration
Abd: soft, non-tender, non-distended, normal bowel sounds
GU: Tanner I female
Extremities: Holds both legs straight while supine. Resists left lower extremity exam and difficult to assess tenderness to palpation; however, there appears to be full passive range of motion of hip, knee, and ankle.
Neuro: Normal reflexes. Sensation appears intact. If forced, will bear weight transiently on left leg.
Skin: No rash.

Labs/Films: None

Impression: Transient Synovitis

Plan: Discharge home with parents, Motrin as needed. Call overnight on-call Pediatrician with concerns or changes.

Friday @ 4am
Olivia’s parents call the practice to report what seems to be worsening pain and she has developed a fever as high as 103 overnight. You are on call tonight and are concerned about a septic arthritis. You decide to send her to the Emergency Department and call ahead to tell the covering physician about her. Please handoff the patient to the very busy Emergency Department Physician.

***Student #1 hands off the above information to Student #2, (6-8 minutes).*** *Do not attempt to report every detail of the information that is given to you. Engage your receiver and ensure that your contingency plan is clear. Try and keep an optimal pace (not too fast or slow).*

**STUDENT #2 -> # 3**

Friday at 2pm. You are the Emergency Department Physician.

You have many patients to see and several who are sick. You note Olivia to be a non-toxic, but fussy child who appears uncomfortable. Olivia has a temperature of 38.9 rectally, blood pressure of 115/70 and pulse to 130. She prefers to hold her leg flexed and externally rotated. She has obvious tenderness on both active and passive movement of her left hip and persistent refusal to walk. You order labs; results are notable for white blood cell count of 15,500, hemoglobin of 11.8, and a platelet count of 400,000. The differential shows 8 bands and 78% neutrophils. Her c-reactive protein is 5.1 mg/dL and the erythrocyte sedimentation rate is 52 mm/hr. The x-ray is read as slight widening of joint space; clinical correlation is suggested. You feel she needs to be admitted for observation due to a possible septic joint.

Please contact the Inpatient Weekend Provider and handoff the patient.

***Student #2 hands off the above information to Student #3, who is called into the room immediately before (6-8 minutes).*** *Do not attempt to report every detail of the information that is given to you. Engage your receiver and ensure that your contingency plan is clear. Try and keep an optimal pace (not too fast or slow).*

**STUDENT #3-> #4**

Sunday @ 11am. You are the Inpatient Weekend Provider.

You are rounding on a large service this morning and have several patients who need your attention. After admission and observation, you are concerned that Olivia’s pain is not better. She is still refusing to walk. Her temperature has been > 38.0 all day today and is not improving. She has been drinking ok, but does not have much of an appetite. Her current exam is notable for continued refusal to move her left lower extremity, with preference for flexion and external rotation. You do not note any cellulitis or erythroderma surrounding her hip.

Your team obtains a hip ultrasound is showing an effusion. Orthopedics is consulted who finds her exam, imaging, and labs concerning for septic arthritis. She is made NPO (non per os) in anticipation of urgent surgical drainage of her joint and you order intravenous antibiotics. When you leave for the day, Olivia is in the operating room.

Please sign out your patient to the Inpatient Weekday Provider. Include any issues you may foresee post-operatively.

***Student #3 hands off the above information to Student #4, who is called into the room directly before (6-8 minutes).*** *Do not attempt to report every detail of the information that is given to you. Engage your receiver and ensure that your contingency plan is clear. Try and keep an optimal pace (not too fast or slow).*

***STUDENT #4 -> #1***

Thursday @ 10am. You are the Inpatient Weekday Provider who has taken care of Olivia for several days in the hospital.

Your team knows Olivia well. She has completed surgical drainage of her joint and has had had many doses of intravenous antibiotics and pain medication while in the hospital. Her drain has been removed and the offending bacteria identified from her wound culture. She is now pain-free and her fevers have resolved. She is walking easily.

She will go home with a long-term intravenous line for 4 weeks of home antibiotics and has supplies arranged and follow-up appointments with Orthopedics and Infectious Disease specialists for the week after hospital discharge. You write her discharge summary and call her Pediatrician.

Please handoff the patient to the Outpatient Pediatrician (Student #1).

***Student #4 hands off the above information to Student #1, who is the original Outpatient Pediatrician (6-8 minutes).*** *Do not attempt to report every detail of the information that is given to you. Engage your receiver and ensure that your contingency plan is clear. Try and keep an optimal pace (not too fast or slow).*
