# Supplementary figures and images for: Team-Based Simulation for Medical Student Handoff Education
Source: MedEdPORTAL. 2016 Oct 21;12:10486. doi: 10.15766/mep_2374-8265.10486 (PMC6440419; doi:10.15766/mep_2374-8265.10486)

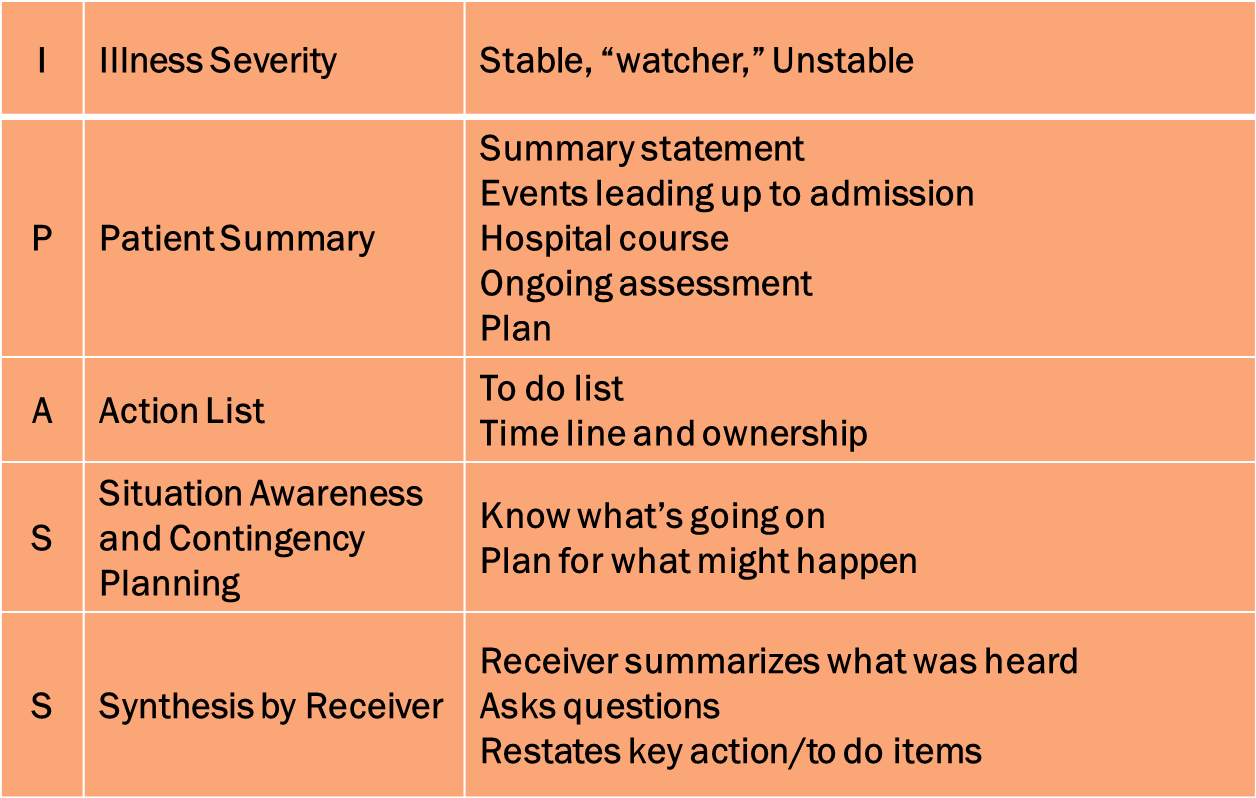


Starner, AJ, et. Al. I-PASS, a Mnemonic to Standardize Verbal Handoffs. Pediatrics 129: 2. 2012

Supplement: Supplementary file 1 — A. Team-Based Simulation for Medical Student Handoff Education.pptx B. Cases.docx C. I-PASS.docx D. Discussion Guide.docx [file mep-12-10486-s001.zip › C. I-PASS.docx]
